# Impact of Levothyroxine Treatment for Hypothyroidism on the Risk of Psychiatric Interventions in Children and Adolescents with Anxiety Disorders: A Retrospective Analysis of Data from the TriNetX Platform

**DOI:** 10.3390/jcm15082893

**Published:** 2026-04-10

**Authors:** Marta Hilmon, Janina Kulińska, Dominik Krzyżanowski, Katarzyna Skórkowska-Telichowska

**Affiliations:** 1 Jerzy Gromkowski Regional Specialist Hospital, 51-149 Wroclaw, Poland; d1.hilmon@gmail.com; 2 Department of Public Health, Faculty of Health Sciences, Wroclaw Medical University, 50-367 Wroclaw, Poland; 3 Department of Clinical Neurosciences, Faculty of Medicine, Wroclaw University of Science and Technology, 50-370 Wroclaw, Poland; dominik.krzyzanowski@pwr.edu.pl; 4 Department of Non-Procedural Clinical Sciences, Faculty of Medicine, Wroclaw University of Science and Technology, 50-370 Wroclaw, Poland; katarzyna.skorkowska-telichowska@pwr.edu.pl

**Keywords:** hypothyroidism, anxiety disorders, children and adolescents, levothyroxine, mental health, psychiatric interventions, suicidal ideation

## Abstract

**Background/Objectives**: Hypothyroidism, including subclinical hypothyroidism, may affect mental health in children and adolescents through disturbances of neurotransmission and dysregulation of the hypothalamic–pituitary–thyroid and stress axes. Anxiety disorders are common in this population and frequently coexist with somatic symptoms overlapping those of hypothyroidism, complicating diagnosis and treatment. This study aimed to evaluate the association between levothyroxine treatment for hypothyroidism and the need for psychiatric interventions in children and adolescents with anxiety disorders. **Methods**: A retrospective cohort study was performed using data from the TriNetX global research network. Patients aged 5–18 years with diagnoses of hypothyroidism (ICD-10: E03) and anxiety disorders (ICD-10: F41) were included. Two propensity score–matched cohorts were analysed: patients treated with levothyroxine (*n* = 1861) and untreated patients (*n* = 1861). Outcomes included psychiatric hospitalisations, use of selective serotonin reuptake inhibitors and tricyclic-like antidepressants, frequency of psychiatric and psychotherapeutic consultations, and the occurrence of suicidal ideation and self-harm. **Results:** Levothyroxine treatment was associated with lower odds of SSRI use (OR = 0.58; *p* < 0.001), fewer psychiatric consultations (OR = 0.48; *p* < 0.001), and lower recorded use of psychotherapy (OR = 0.75; *p* = 0.029). Suicidal ideation and self-harm were recorded less frequently in the treated group (OR = 0.53; *p* = 0.001). No significant differences were observed in psychiatric hospitalisation rates. Use of tricyclic-like antidepressants was uncommon and did not differ significantly between groups. **Conclusions**: Among children and adolescents with comorbid anxiety disorders, levothyroxine treatment for hypothyroidism is associated with lower recorded utilization of certain psychiatric services and lower recorded rates of suicidal ideation and self-harm. Due to the retrospective design, causal inferences cannot be made, and the findings should be considered hypothesis-generating, requiring confirmation in prospective studies with standardised psychiatric outcome measures.

## 1. Introduction

Hypothyroidism, whether primary, secondary, or subclinical, affects mental functioning, particularly during development [1,2]. Thyroid hormones such as thyroxine (T4) and triiodothyronine (T3) play a key role in neuroplasticity, central nervous system (CNS) myelination, regulation of the stress axis, and modulation of neurotransmitter systems in the brain [3,4]. During childhood and adolescence, hormonal disorders can disrupt neurobiological balance, making individuals more susceptible to emotional disorders, including anxiety disorders [5].

Anxiety disorders are among the most commonly diagnosed mental health problems in children and adolescents. The symptoms can be non-specific, encompassing psychological symptoms as well as physical complaints such as abdominal pain, muscle tension, and tachycardia [6]. These symptoms often overlap with the manifestations of hypothyroidism, complicating differential diagnosis and potentially leading to delays in appropriate therapeutic intervention.

Studies have shown that thyroid hormone deficiency can disrupt the balance of neurotransmitters such as serotonin, γ-aminobutyric acid (GABA), and norepinephrine, which are involved in anxiety mechanisms [7]. Furthermore, disorders of the HPT (hypothalamic–pituitary–thyroid) axis affect the functioning of the HPA (hypothalamic–pituitary–adrenal) axis, the hyperactivity of which is typically observed in anxiety disorders [8].

Despite the growing number of research findings on the neurobiological links between hormone levels and mental health, large-scale population studies examining the impact of hypothyroidism on anxiety disorders in children and adolescents remain scarce. The available studies are mostly limited to individual case studies or small clinical trials [1,9].

In light of these limitations, analyses using large databases such as TriNetX enable the relationship between hypothyroidism and anxiety disorder occurrence to be assessed in large, clinically diverse paediatric cohorts. Although retrospective evaluation has obvious limitations, these analyses provide important and valuable insights.

Anxiety disorders represent a heterogeneous clinical group, and available evidence suggests that thyroid dysfunction has most consistently been linked to generalized anxiety disorder (GAD) and panic disorder (PD) [10], with comparatively limited data for social phobia/social anxiety disorder. Adolescence may further modify this association given dynamic neuroendocrine changes and well-established sex differences in anxiety prevalence that become more pronounced after puberty [11,12]. Developmental factors should also be considered when interpreting these relationships. Anxiety is highly comorbid in neurodevelopmental conditions, including autism spectrum disorder (ASD) and intellectual disability (ID); meta-analytic estimates suggest that approximately ~40% of children with ASD meet the criteria for at least one anxiety disorder, with wide variability across studies [13]. In real-world datasets, ASD/ID can be identified using ICD codes, enabling descriptive reporting of these comorbidities and sensitivity analyses (e.g., adjustment/matching including ASD/ID, or analyses excluding ASD/ID).

Although thyroid dysfunction has also been widely associated with mood disorders, particularly depressive symptoms, anxiety disorders are highly prevalent in paediatric populations and often represent an early clinical manifestation of emotional dysregulation. In the present study, anxiety disorders were examined separately in order to avoid conflating anxiety-related outcomes with depressive symptomatology and to more precisely explore the relationship between hypothyroidism and anxiety-related psychiatric outcomes. While mood and anxiety disorders frequently co-occur in children and adolescents and may share overlapping biological mechanisms related to thyroid hormone dysregulation, analysing anxiety disorders as a distinct outcome may provide additional insight into early psychiatric vulnerability associated with thyroid dysfunction.

The aim of the study is to assess the relationship between the treatment of hypothyroidism and recorded psychiatric interventions associated with anxiety disorders in children and adolescents aged 5–18 years, with particular emphasis on the impact of levothyroxine treatment on the frequency of psychiatric interventions. The analysis includes the risk of starting pharmacotherapy with selective serotonin reuptake inhibitors (SSRIs) and tricyclic antidepressants (TCAs), the need for psychotherapy, the need for hospitalization in psychiatric wards, the need for diagnostic evaluation and treatment by a psychiatrist in consultation, and the occurrence of suicidal thoughts and self-harm.

A further objective is to establish whether levothyroxine treatment is associated with differences in recorded psychiatric care utilisation in this age group, and whether endocrinological diagnostics should be incorporated into routine assessments of children and adolescents presenting with anxiety symptoms.

## 2. Materials and Methods

The analysis was conducted using the TriNetX Global Collaborative Network platform, which integrates electronic medical record data from 147 healthcare centres worldwide. A retrospective cohort study was conducted involving children and adolescents aged 5–18 years at the time of hypothyroidism diagnosis (ICD-10: E03) alongside coexisting anxiety disorders (ICD-10: F41).

Age was defined as the patient’s age at the index event. The cohort was restricted to individuals aged 5–18 years at the time of cohort entry using the “Age at Event” filter within the TriNetX platform.

No psychiatric comorbidities were excluded from the analysis. This decision was made deliberately to preserve ecological validity and to reflect the real-world clinical complexity of paediatric anxiety disorders. In routine clinical practice, anxiety disorders in children and adolescents frequently coexist with depressive symptoms and neurodevelopmental conditions. Excluding such comorbidities would have resulted in an artificially restricted cohort and reduced the generalizability of the findings. The aim of the study was not to isolate anxiety disorders independent of other psychiatric diagnoses, but rather to evaluate whether levothyroxine treatment in patients diagnosed with anxiety disorders and subclinical hypothyroidism is associated with differences in subsequent psychiatric outcomes within a real-world population.

During the preparation of this manuscript, the authors used the AI tool MediChat (Medico PZWL) to assist with the preliminary literature review. The obtained materials were subsequently reviewed and edited by the authors.

Cohort definitions:

Cohort 1 (N = 1861): Patients who started levothyroxine treatment after being diagnosed with hypothyroidism and anxiety disorders.

Cohort 2 (N = 1861): Patients diagnosed with hypothyroidism and comorbid anxiety disorders who did not receive hormone therapy.

Patients receiving psychiatric pharmacotherapy (e.g., antidepressants, antipsychotics, central nervous system stimulants or antiepileptic drugs) during the index period were excluded from both groups.

To ensure comparability between the groups, propensity score matching (PSM) was used to take demographic and clinical data into account. After matching, both cohorts consisted of 1861 patients, balanced in terms of age and gender. Propensity score matching (PSM) was performed using age and sex as covariates within the TriNetX platform. Matching was conducted using a 1:1 nearest-neighbour greedy algorithm without replacement. Balance between cohorts after matching was assessed using standardized mean differences (SMDs), with values <0.1 considered indicative of adequate balance.

Index event and observation period:

The index event was defined as the moment when the inclusion criteria for the cohort were met, i.e., the first simultaneous diagnosis of anxiety disorders and hypothyroidism.

The index date was defined according to the TriNetX “index event” construct as the first date on which all cohort-defining criteria were fulfilled. In the levothyroxine-treated cohort, this required a recorded diagnosis of hypothyroidism (ICD-10: E03), a diagnosis of anxiety disorders (ICD-10: F41), and the documentation of levothyroxine use (RxNorm: 10582). In the untreated cohort, the index date corresponded to the first date on which the diagnostic criteria (ICD-10: E03 and F41) were fulfilled without documentation of levothyroxine use.

The observation period was five years (1825 days) from the index date. Patient data were analysed until the occurrence of a given event, the end of the observation period, or the last recorded medical entry.

The range of dependent variables analysed included hospitalisation in a psychiatric ward, treatment with selective serotonin reuptake inhibitors (SSRIs) and tricyclic antidepressants (TCAs), the need for psychotherapy sessions, and psychiatric diagnoses in the form of consultations. The occurrence of suicidal thoughts and self-harm was also analysed (ICD-10: R45.851, X71–X83).

Outcome definitions

Hospitalisation was defined using the TriNetX concept set “Subsequent Hospital Inpatient or Observation Care” (CPT 1013668) or “Visit: Inpatient Encounter”, representing any subsequent inpatient or observation-level hospital encounter without restriction to specific diagnostic categories. Selective serotonin reuptake inhibitors (SSRIs) were defined using the ATC classification code N06AB. Psychotherapy sessions were defined using the CPT code 1021137 (“Psychotherapy Services and Procedures”) within the TriNetX platform. Identification relied on documented procedure codes recorded in participating healthcare systems. The composite endpoint of suicidal ideation and self-harm was defined using ICD-10-CM codes R45.851 (suicidal ideation) and X71–X83 (intentional self-harm). Tricyclic antidepressants (TCAs) were defined using the VA drug class CN601 (“Tricyclic Antidepressants”) within the TriNetX platform. Psychiatric consultations were defined using CPT code 90791 (“Psychiatric diagnostic evaluation”), representing documented psychiatric diagnostic encounters rather than incident psychiatric diagnoses.

Analysed outcomes

The range of dependent variables analysed included subsequent hospital inpatient or observation care, treatment with selective serotonin reuptake inhibitors (SSRIs) and tricyclic antidepressants (TCAs), the need for psychotherapy sessions, and psychiatric diagnostic consultations. The composite endpoint of suicidal ideation and self-harm was analysed (ICD-10: R45.851, X71–X83).

Statistical analysis

A comparative analysis was conducted of the incidence and risk of selected psychiatric events among children and adolescents with hypothyroidism and coexisting anxiety disorders. The following outcome variables were included: hospitalisations in psychiatric wards (inpatient/observation care), use of psychotropic drugs (SSRIs and tricyclic antidepressants—TCAs), composite endpoint of suicidal and self-harm (ICD-10: R45.851, X71–X83), use of psychotherapy, and psychiatric diagnosis.

For each endpoint, the following were calculated: incidence (risk), difference in risk between groups, relative risk (RR), odds ratio (OR), 95% confidence intervals (95% CI), *p*-values for significance tests (*t*-test or χ^2^ test). In addition, survival analysis (Kaplan–Meier) was performed using the log-rank test and calculating the hazard ratio (HR) and assessing the proportionality of risks. The analysis was supplemented with the number of instances of each event, i.e., the total number of occurrences of a given event during the observation period. For comparisons between cohorts in this regard, Student’s *t*-test for independent samples was used, taking into account the means, standard deviations, and medians of the number of cases.

All analyses were performed on cohorts matched using propensity score matching, over a five-year observation period (1825 days) from the index date.

Levothyroxine treatment was associated with lower odds of recorded psychiatric diagnostic consultations, psychotherapy use, SSRI use, and suicidal ideation or self-harm, while no significant difference was observed for psychiatric hospitalisations. These findings provide a quantitative overview of the recorded healthcare utilisation outcomes analysed in this study.

## 3. Results

### 3.1. Study Population and Baseline Characteristics

The analysis included two cohorts of 1861 patients each. All patients were aged 5–18 years at the time of study eligibility and were matched by propensity score matching for age and gender. Over a five-year follow-up period, the incidence of psychiatric interventions and the characteristics of events were compared in the levothyroxine-treated cohort (Hypo + Levo + Anxiety) and the untreated cohort (Hypo − Levo + Anxiety). Baseline demographic characteristics of the study population before and after propensity score matching are presented in Table 1 and Table 2.

Before matching, minor differences in age and sex distribution were observed between cohorts, whereas after matching, both groups were well-balanced, with minimal standardised differences. This indicates good balance between cohorts for the matched variables (age and sex).

### 3.2. Psychiatric Outcomes

Table 3 summarises the comparative risks of psychiatric outcomes in levothyroxine-treated and untreated patients over the five-year follow-up period. Levothyroxine treatment was associated with lower odds of psychiatric diagnosis, psychotherapy, SSRI use, and suicidal ideation or self-harm, while no significant difference was observed for psychiatric hospitalisations. These findings provide a quantitative overview of the main clinical outcomes analysed in this study.

No significant differences were observed in psychiatric hospitalisations between the study groups. Across the remaining analysed outcomes, a lower frequency of events was observed in the levothyroxine-treated group, including psychiatric diagnoses, psychotherapy use, SSRI treatment, and the composite endpoint of suicidal ideation and self-harm. A similar pattern was noted for tricyclic antidepressant (TCA) use; however, this finding should be interpreted with caution due to the relatively low number of events (Table 3).

With the exception of psychiatric hospitalisations, where no differences were found between the study groups, and the use of TCAs, where differences were found in favour of less frequent use of this treatment in the hormone-treated group but without achieving statistical significance, all analysed psychiatric interventions, including psychiatric diagnosis, psychotherapy, SSRI use and the occurrence of suicidal thoughts and self-harm, were recorded less frequently in the group of patients treated with levothyroxine.

## 4. Discussion

The results obtained indicate that levothyroxine treatment was associated with lower recorded rates of psychiatric interventions related to anxiety disorders in children and adolescents. Specifically, lower recorded rates of SSRI use, psychiatric evaluations, psychotherapy sessions, the composite endpoint of suicidal ideation and self-harm was observed in the treated group than in the untreated group. These findings may reflect differences in recorded healthcare utilization patterns rather than direct evidence of clinical improvement [14,15].

The observed association between levothyroxine treatment and reduced anxiety-related psychiatric interventions is consistent with a potential pathophysiological link between thyroid hormone signalling and anxiety symptomatology. While causal inference cannot be definitively established in a retrospective design, the directionality and consistency of findings across multiple psychiatric endpoints support the hypothesis that subclinical hypothyroidism may contribute to anxiety vulnerability in paediatric populations and that thyroid hormone normalisation may modify this trajectory.

There is limited literature on the co-occurrence of hypothyroidism and anxiety disorders in the paediatric population, and studies conducted in the adult population show a significant association between thyroid hormone deficiency and the severity of anxiety symptoms, irritability, and emotional dysregulation [16]. In this patient group, levothyroxine treatment was often associated with improved mental functioning, which may be an important reference point when interpreting results in children and adolescents [17,18].

Potential biological mechanisms include disturbances in the functioning of the hypothalamic–pituitary–thyroid (HPT) axis, affecting the limbic and serotonergic systems—structures that play a key role in processing emotions and regulating anxiety [7,19]. T3 and T4 deficiency can disrupt the functioning of the hippocampus and amygdala, leading to increased emotional reactivity. Additionally, thyroid hormones modulate the activity of the hypothalamic–pituitary–adrenal (HPA) axis [8], which is typically hyperactive in anxiety disorders. Due to the intense development of the central nervous system and high neuroplasticity in children and adolescents, the impact of these disorders can be particularly significant.

According to the results obtained, the group of patients treated with levothyroxine had lower recorded rates of psychiatric interventions. These findings may reflect differences in healthcare utilisation patterns rather than direct measures of anxiety symptom severity.

The absence of differences in psychiatric hospitalisations despite differences in outpatient psychiatric interventions may reflect the fact that hospitalisation represents a more severe and relatively rare endpoint, typically associated with acute psychiatric crises rather than patterns of outpatient care utilisation.

All analyses were performed on cohorts that were matched using propensity score matching over a five-year (1825-day) observation period from the index date. This long time horizon was adopted to capture the short- and long-term effects of levothyroxine treatment in children and adolescents with hypothyroidism and coexisting anxiety disorders. This period allows us to assess the stability of observed associations, which is important because the impact of hormone treatment may become apparent gradually as thyroid function normalises and neurobiological processes adapt. The period also takes into account growth and development, including puberty, which can modify the severity of anxiety symptoms and the response to treatment. A longer observation period minimises the risk of overlooking delayed events such as psychiatric hospitalisations, the initiation of psychotherapy or pharmacotherapy. It also increases the reliability of survival analysis, enabling more accurate modelling of the Kaplan–Meier curve and calculation of the hazard ratio. The choice of a five-year observation period aligns with the approach taken in observational studies within psychiatry and paediatric endocrinology. In these fields, the effects of interventions may only become apparent after several years, and shorter time intervals could result in an underestimation of the actual impact of treatment.

## 5. Conclusions

The results of the analyses suggest that levothyroxine treatment in children and adolescents with comorbid anxiety disorders was associated with lower recorded utilisation of certain psychiatric services compared to patients who did not receive hormone treatment, the treated group had lower recorded rates of SSRIs, fewer psychiatric evaluations, less need for psychotherapy, and fewer reports of suicidal thoughts and self-harm. While no significant differences in psychiatric hospitalisations were observed, the other endpoints indicate differences in recorded psychiatric care utilisation between the analysed groups.

These findings should be interpreted cautiously, as they reflect associations observed in routinely collected healthcare data rather than direct measures of clinical improvement or symptom severity.

The results obtained are clinically significant: they may indicate differences in healthcare utilisation patterns rather than direct modification of the course of mental disorders. The reduced frequency of antidepressant use (including SSRIs and TCAs) may reflect differences in prescribing patterns, healthcare utilisation, follow-up intensity, or unmeasured confounding rather than differences in disorder severity. Similarly, the lower frequency of suicidal thoughts and the reduced need for psychotherapeutic interventions may reflect differences in recorded clinical management, access to care, or other unmeasured factors.

A key conclusion of the study is therefore that the findings highlight the potential relevance of considering thyroid function assessment in children and adolescents presenting with anxiety disorders; however, the present results should not be interpreted as establishing clinical recommendations.

These findings may also highlight the importance of integrated care approaches in paediatric populations, including closer collaboration between paediatricians and mental health professionals, to facilitate earlier identification and comprehensive evaluation of children presenting with anxiety symptoms and possible endocrine comorbidities. Earlier recognition of thyroid dysfunction in such cases may help guide appropriate treatment and, in some patients, potentially reduce the need for psychiatric pharmacotherapy.

Further prospective evaluation of the issue is planned, and additional studies may be necessary to incorporate such recommendations into the standards of practice for medical practitioners.

Due to the limitations of this study resulting from the chosen research method, further prospective and controlled analyses are necessary in order to make a clear assessment of the impact of levothyroxine supplementation on anxiety disorders in children and adolescents with hypothyroidism.

## 6. Study Limitations

The main limitation of this analysis is the limited availability and completeness of thyroid function laboratory data, such as TSH, FT4, and FT3 concentrations. This makes it impossible to clearly identify the type and severity of hypothyroidism, as well as the degree of euthyroidism (i.e., the effectiveness of hormone treatment). Patient selection was based solely on ICD-10 classification codes, reflecting a limitation of the database used.

Due to the lack of an unambiguous ICD-10 code that allows for differentiation between the different types of hypothyroidism, the analysis included all patients marked with code E03, which covers primary hypothyroidism of various causes, including both subclinical and overt forms. While this code is commonly used in population studies, it does not permit the accurate differentiation of clinical phenotypes, which could lead to heterogeneity in the analysed sample.

The TriNetX data structure also does not permit the inclusion of information on the dosage and duration of hormonal and psychiatric treatment, medication adherence, or detailed clinical characteristics of comorbid mental, somatic, or neurodevelopmental disorders. 

The TriNetX Global Collaborative Network integrates electronic health record data from multiple healthcare organisations across several countries rather than a single national healthcare system. Consequently, the database does not provide standardised individual-level socioeconomic variables such as parental education, household income, or insurance status across participating institutions. Socioeconomic factors and healthcare access may influence patterns of healthcare utilisation and mental health service use; however, these variables could not be consistently assessed in the present analysis. In addition, differences in healthcare systems, referral pathways, and access to specialist services between countries represented in the TriNetX network may contribute to variability in recorded psychiatric interventions. Cultural attitudes toward mental health care, regional differences in healthcare access (e.g., urban versus rural settings), and other social determinants of health may further influence patterns of diagnosis and treatment but could not be evaluated within the available dataset. 

This limits the ability to fully control the potential confounding factors and to interpret the observed associations. In addition, the observational nature of this study introduces the possibility of confounding by indication. Patients receiving levothyroxine may have differed systematically from untreated patients with respect to disease severity, clinical decision-making processes, or patterns of healthcare utilisation that were not fully captured in the available database variables. The analysed outcomes primarily represent recorded healthcare utilisation and clinical interventions, rather than direct measures of anxiety symptom severity. Therefore, the observed differences between cohorts may also reflect variation in healthcare access, follow-up intensity, clinician practice patterns, or residual unmeasured confounding. Propensity score matching was limited to age and sex; therefore, residual confounding related to baseline psychiatric burden, healthcare utilization patterns, and other unmeasured clinical characteristics cannot be excluded.

The analysis did not adjust for specific psychiatric comorbidities, including depressive disorders and neurodevelopmental conditions such as autism spectrum disorders or intellectual disability. These conditions frequently coexist with anxiety disorders in paediatric populations and may influence psychiatric service utilisation and clinical outcomes. Due to limitations inherent to the TriNetX database structure, detailed stratified analyses based on psychiatric comorbidity severity or developmental phenotype were not feasible. Future studies should investigate whether the observed associations differ across specific psychiatric subgroups.

As this outcome was analysed as a composite endpoint, suicidal ideation and self-harm were not evaluated separately. These components may differ in clinical meaning and coding practices, which limits the granularity of the analysis and warrants cautious interpretation. We acknowledge that psychotherapy services may be variably captured across healthcare systems due to differences in coding practices and documentation standards, which may introduce differential ascertainment bias across healthcare systems. Analyses were conducted across multiple endpoints without formal adjustment for multiple comparisons. Therefore, the findings should be interpreted with caution, particularly for results close to the statistical significance threshold. The lack of a statistically significant difference in psychiatric hospitalisations should be interpreted with caution. Hospitalisation represents a relatively rare and more severe clinical endpoint, and its occurrence may be strongly influenced by local clinical practices and healthcare system organisation. In addition, the broad definition of hospitalisation used in the TriNetX database (including both inpatient and observation-level encounters) may have limited the ability to detect subtle differences between the analysed groups. It was also impossible to assess the reasons for initiating or discontinuing levothyroxine treatment. Therapeutic decisions may have been based on unrecorded factors, such as caregiver preferences, the availability of specialists or the severity of clinical symptoms. Additionally, some patients may have become adults during the five-year follow-up period, a factor that should be considered when interpreting long-term results.

## Figures and Tables

**Table 1:** Baseline demographic characteristics of the study cohorts before propensity score matching.

Cohort	Characteristic	Mean ± SD	*n*	% of Cohort	*p*-Value	Std. Diff.
1	Age at index date (years)	17.3 ± 7.2	1867	100.0	0.047	0.046
2	Age at index date (years)	17.0 ± 5.9	11,359	100.0	—	—
1	Female	—	1404	75.2	0.064	0.047
2	Female	—	8310	73.2	—	—
1	Male	—	435	23.3	0.055	0.049
2	Male	—	2883	25.4	—	—

**Table 2:** Baseline demographic characteristics of the matched cohorts after propensity score matching.

Cohort	Characteristic	Mean ± SD	*n*	% of Cohort	*p*-Value	Std. Diff.
1	Age at index date (years)	17.1 ± 6.8	1861	100.0	0.891	0.004
2	Age at index date (years)	17.1 ± 6.6	1861	100.0	—	—
1	Female	—	1398	75.1	0.879	0.005
2	Female	—	1402	75.3	—	—
1	Male	—	435	23.4	0.877	0.005
2	Male	—	431	23.2	—	—

Abbreviations: SD, standard deviation; Std. diff., standardized difference. Cohort 1: levothyroxine-treated patients; Cohort 2: untreated patients. Percentages may not total 100% due to rounding.

**Table 3:** Psychiatric outcomes in anxiety disorders by levothyroxine treatment status.

Outcomes	Treated Group (%)	Untreated Group (%)	OR (95% CI)	*p*-Value
Psychiatric hospitalisations	15.1	15.4	0.975 (0.816–1.166)	0.784
Psychiatric diagnosis	2.5	5.1	0.482 (0.338–0.687)	<0.001
Psychotherapy	6.1	7.9	0.754 (0.585–0.972)	0.029
Suicidal ideation/self-harm	2.5	4.6	0.535 (0.373–0.768)	0.001
Use of SSRIs	12.0	19.1	0.580 (0.484–0.696)	<0.001
Use of TCAs	1.0	1.8	0.554 (0.315–0.975)	0.038

## Data Availability

The data used in this study were derived from the TriNetX database and are not publicly available due to data protection and licensing restrictions. Aggregated data supporting the findings may be made available from the corresponding author upon reasonable request.
